# Revisiting the Gateway Drug Hypothesis for Cannabis: A Secondary Analysis of a Nationwide Survey Among Community Users in Japan

**DOI:** 10.1002/npr2.70033

**Published:** 2025-07-01

**Authors:** Yuji Masataka, Munenori Katayama, Futaba Umemura, Takeshi Sugiyama, Naoko Miki, Yoshiyuki Akahoshi, Chihiro Oka, Takashi Asahi, Takashi Matsumori, Ichiro Takumi, Hidetoshi Murata, Toshihiko Matsumoto

**Affiliations:** ^1^ Department of Neurosurgery St. Marianna University School of Medicine Kawasaki Japan; ^2^ General Incorporated Association Green Zone Japan Saitama Japan; ^3^ Department of Neurology Kumamoto Seijo Hospital Kumamoto Japan; ^4^ Department of Drug Dependence Research National Institute of Mental Health, National Center of Neurology and Psychiatry Tokyo Japan; ^5^ Graduate School of Medical and Dental Sciences, Niigata University Niigata Japan; ^6^ College of Bioresource Sciences, Nihon University Fujisawa Japan; ^7^ Department of Neurosurgery Yokohama City University, Graduate School of Medicine Yokohama Japan; ^8^ Department of Neurosurgery Kanazawa Neurosurgical Hospital Nonoichi Japan

**Keywords:** cannabis, gateway drug hypothesis, Japan, marijuana, substance use

## Abstract

**Aim:**

Cannabis has historically been used for medicinal and industrial purposes, but is strictly regulated worldwide due to the psychoactive effects of THC. In Japan, cannabis is frequently labeled a “gateway drug,” yet strong causal evidence for progression to other substances is limited. This study investigates whether cannabis acts as a gateway drug among Japanese users.

**Methods:**

An anonymous online survey was conducted in January 2021 with 3900 individuals reporting lifetime cannabis use in Japan. Participants were recruited via social media. The survey gathered data on demographics, cannabis and other substance use history, order of substance initiation, psychiatric background, and criminal records. A Sankey diagram visualized substance use progression, and odds ratios were calculated to assess the likelihood of using other substances following cannabis use.

**Results:**

Of all respondents, 81.5% were male, with the largest age group being 20–24. Tobacco and alcohol were the most common initial substances, while cannabis was typically the third. Odds for subsequent use of alcohol, tobacco, methamphetamine, and other illicit drugs after cannabis use were 1.25, 0.77, 0.08, and 0.78, respectively, suggesting low probabilities of progression. Nearly half of those who reported cannabis as their third drug did not use other substances afterward.

**Conclusion:**

Cannabis use in Japan typically follows alcohol and tobacco, and rarely leads to further drug use. These findings challenge the gateway hypothesis in the Japanese context. Shared vulnerabilities and strict drug policies may shape these patterns. Further research is warranted to explore the impact of legal changes on drug use behavior.

**Registry and Registration Number of the Study/Trial:**

Not applicable.

## Abstract 2

This study reexamines the gateway drug hypothesis for cannabis using data from 3900 community‐based users in Japan. Findings show that cannabis is typically preceded by alcohol and tobacco and rarely leads to methamphetamine or other illicit drug use, challenging the hypothesis in the Japanese context. Sociocultural and structural factors, rather than cannabis itself, may better explain subsequent substance use patterns.

## Graphical abstract

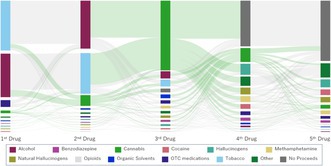

## 1 Introduction

*Cannabis sativa* Linne, an annual plant native to Central Asia, has long been cultivated worldwide. In Japan, its fibers and seeds have been traditionally used for purposes such as making sacred shrine ropes and as ingredients in spice blends such as shichimi togarashi [1]. Cannabis contains more than 160 bioactive compounds collectively known as cannabinoids [2]. Cannabinoids exhibit various pharmacological effects, including analgesic [3], anticonvulsant [4], and anti‐inflammatory [5] actions, and have been recorded as medicinal agents since ancient times [6]. Among these cannabinoids, Δ9‐tetrahydrocannabinol (THC) is the most abundant and is known for its characteristic psychoactive effects [7]. Due to these properties, cannabis was classified as a Schedule IV substance under the 1961 Single Convention on Narcotic Drugs and has been regulated globally as a highly addictive drug [8].

The assessment of cannabis‐related health risks has changed over time depending on sociocultural and historical contexts. In the early 20th century, international regulation became stricter, influenced by antimarijuana campaigns, especially in the United States, being driven more by political and social factors than by scientific evidence [9, 10]. Since the 1990s, research has revealed the existence of endogenous cannabinoids and their receptors and related enzymes in the human body, known collectively as the endocannabinoid system (ECS) [11]. This discovery has positioned the ECS as a target for the treatment of various disorders. Consequently, countries such as the United States, Canada, and parts of Europe have expanded the legalization of medical cannabis, prompting a reevaluation of cannabis' risks and benefits based on scientific data. Recent studies have suggested that cannabis is somewhat less harmful than legal substances such as alcohol and tobacco [12].

Although cannabis is often regarded as less harmful than other illicit substances, concerns remain about its potential role in facilitating progression to the use of more dangerous drugs. This concern is encapsulated in the so‐called “gateway drug hypothesis,” which originated from epidemiological and longitudinal studies conducted primarily in the United States since the 1960s. The hypothesis posits that individuals who begin using cannabis are at greater risk of subsequently engaging in the use of harder drugs, such as cocaine or heroin [13, 14]. Numerous studies supporting this hypothesis have consistently shown that cannabis users report higher rates of subsequent illicit drug use compared to nonusers, with earlier initiation of cannabis use further increasing this likelihood [13, 14]. However, the majority of these studies are correlational in nature and fall short of establishing a direct causal relationship. They merely indicate that a proportion of cannabis users also engage in other drug use, without sufficiently accounting for key confounding factors such as social, psychological, and environmental influences, or individual personality traits.

Animal studies have also suggested that exposure to cannabinoids may alter the brain's reward system, potentially enhancing sensitivity or tolerance to other substances [15, 16, 17]. Nonetheless, the extent to which these findings can be generalized to humans remains contentious, given the complexity of human behavior and environmental factors.

Alternative viewpoints suggest that individuals who initiate cannabis use at a young age may already possess underlying vulnerabilities, such as risk‐taking personality traits, adverse family environments, or social networks where polysubstance use is common [18, 19, 20]. These factors may independently contribute to the observed association between cannabis use and subsequent engagement in other drug use.

Despite these limitations, the gateway drug hypothesis continues to shape drug policy worldwide, including in Japan. The Japanese government has cited this hypothesis as a key rationale for reinforcing cannabis regulations. Prior to the 2023 amendment of the Cannabis Control Act, which introduced criminal penalties for cannabis use [21], the Ministry of Health, Labour and Welfare (MHLW) presented materials referencing this hypothesis during expert panel discussions [22]. These materials drew on findings from a Ministry of Justice report (White Paper on Crime, 2020) [23], which showed that approximately half of stimulant offenders had a history of prior cannabis use, with many initiating cannabis use before the age of 20.

Importantly, this government report focused exclusively on a population of incarcerated individuals detained under Japan's Stimulants Control Law, limiting the generalizability of the findings to broader drug‐using populations. Moreover, while cannabis was reported as the most common “first drug” among offenders under 30 years old (42.6%), organic solvents were the most common first substance among offenders aged 30–49, and methamphetamine was most common in those aged 50 and older. Despite this, the focus was primarily placed on the under‐30 group, while the trends observed in older cohorts were largely disregarded.

Another critical limitation is that tobacco and alcohol—known common precursors to cannabis use—were excluded from the survey's list of substances, thereby preventing a full understanding of the true initiation patterns. While these findings have been cited to support the narrative of cannabis as a gateway drug, the methodological constraints and selective interpretation of data raise questions regarding the robustness of this claim in the Japanese context.

Against this backdrop, we conducted a secondary analysis of nationwide online survey data from cannabis users in Japan, aiming to critically reexamine the validity of the gateway drug hypothesis in this context. Specifically, we sought to clarify the patterns and sequences of cannabis and other drug use among community‐based users.

## 2 Methods

### 2.1 Survey Procedure

This study utilized an anonymous self‐administered online questionnaire via Google Forms. Recruitment targeted community‐based cannabis users in Japan. From January 21 to February 3, 2021, we posted survey information and a URL link to the questionnaire on various media platforms managed by the authors, including a blog, YouTube channel, email newsletters, X (formerly Twitter), and Instagram accounts. These platforms are operated by Green Zone Japan, a nonprofit organization advocating for medical cannabis in Japan and leading discussions on cannabis policy reform. This study was conducted in accordance with the “Declaration of Helsinki” and the “Ethical Guidelines for Medical Research Involving Human Subjects.” The study was approved by the Ethics Review Board of the National Center of Neurology and Psychiatry (Approval Number: A2020‐117; Approval Date: January 8, 2021).

### 2.2 Participants

The inclusion criteria were as follows: (1) Japanese nationals who self‐identified as Japanese; and (2) individuals reporting lifetime cannabis use (i.e., those who had used cannabis at least once). Exclusion criteria included: (1) participants who did not consent to the survey; (2) respondents who indicated it was a duplicate entry; (3) respondents currently residing outside Japan; (4) respondents who reported no history of cannabis use; and (5) respondents who reported no experience with any drugs other than cannabis. Additionally, participants whose responses to drug use sequencing contained clear inconsistencies (e.g., identical answers for both the first and second substances, or skipping the second substance but providing a third) were excluded from the analysis.

### 2.3 Measures

The questionnaire collected data on:

- Demographic information (gender, age, nationality, place of residence);
- Social history (educational background, employment status);
- Criminal history (drug‐related and other offenses);
- Clinical genetic factors (family history of mental disorders, substance use disorders, addictive behaviors, and suicide);
- Cannabis use patterns (age at first use, recent use, experience with THC‐concentrated products such as hashish or wax, and psychiatric treatment history related to cannabis use);
- Experience and order of use of other substances;
- Cannabis‐related mental health effects;
- Psychiatric history, self‐reported diagnoses, and timing of diagnosis;
- History of suicidal ideation or suicide attempts.

For item vi, participants were asked: “Please select all substances (other than cannabis) that you have ever used (excluding appropriate medical use),” with the following options:

– Tobacco
– Alcohol
– Methamphetamine
– Organic solvents
– Cocaine
– Natural hallucinogens (e.g., psilocybin mushrooms, ayahuasca, peyote)
– Hallucinogens (e.g., LSD, MDMA)
– Opioids (e.g., heroin, morphine, opium, medical narcotics)
– Benzodiazepine‐based hypnotics
– Over‐the‐counter (OTC) medications (e.g., cough suppressants)
– None of the above

Participants were then asked to rank up to five substances (including cannabis) in the order they were first used.

### 2.4 Statistical Analysis

Following precedents in previous studies [24], we visualized the sequences of substance use from the first to the fifth substance using a Sankey diagram, which displays the relationship between nodes (substances) and links (transitions between substances) proportionally based on response counts. This method offers an intuitive way to display both the direction and magnitude of transitions in a single figure, making it especially useful for capturing complex behavioral patterns that cannot be well understood by using traditional charts or tables. In recent medical research, Sankey diagrams have been applied to visualize prognosis and disease progression over time [25, 26]. In the present study, we collected data on the sequence of use for multiple substances, including cannabis, and considered this visualization method particularly suitable for illustrating such patterns.

Additionally, we conducted a series of multivariable logistic regression analyses to examine the association between individual characteristics and the likelihood of transitioning to the following four substance categories after cannabis use: alcohol, tobacco, methamphetamine, and any illegal drugs (defined as substances other than tobacco, alcohol, benzodiazepines, and OTC medications). Samples for each regression analysis were extracted by excluding the respondents who experienced the substance of concern prior to cannabis use. The dependent variable in each model was a binary indicator of transitioning to each substance category after marijuana use. Independent variables included gender, age group, highest education, employment status, and history of mental health disorders. Categorical predictors were dummy coded, with the following reference categories: “Female” for gender, “Under 19” for age, “Junior high school” for education, “Employed” for employment status, and “No” for history of mental disorders. Categories with fewer than 10 observations were considered low frequency. Depending on the nature of the variable, such categories were either removed from the analysis or combined with similar categories to ensure model stability and interpretability. All models were adjusted for these covariates simultaneously. Adjusted odds ratios (ORs) and their 95% confidence intervals (CIs) were estimated to quantify the strength and direction of associations. The Hosmer and Lemeshow goodness‐of‐fit test was conducted to analyze the fit of each model.

All analyses were conducted using R version 4.3.2, with the Sankey diagrams created using the networkD3 package. Statistical significance was determined at the 0.05 level.

## 3 Results

### 3.1 Participant Characteristics

A total of 4795 individuals responded to the survey during the study period. Of these, 895 were excluded based on the exclusion criteria, resulting in 3900 participants included in the final analysis. The profiles of the 3900 respondents included in the analysis are shown in Table 1. Among them, 3180 (81.5%) were male. The most common age group was 20–24 years (1127 respondents, 28.9%). Regarding education, the most frequent response was high school graduation (1632 respondents, 41.8%). As for the use of substances other than cannabis, alcohol was the most common (3755 respondents, 96.3%), followed by tobacco (3657 respondents, 93.8%).

**TABLE 1:** Participant characteristics (*N* = 3900).

Category	*N* (%)
Gender—Male	3180 (81.5)
Gender—Female	697 (17.9)
Gender—Other	23 (0.6)
Age—Under 15	3 (0.1)
Age—15–19	274 (7.0)
Age—20–24	1127 (28.9)
Age—25–29	656 (16.8)
Age—30–34	579 (14.8)
Age—35–39	496 (12.7)
Age—40–44	399 (10.2)
Age—45–49	169 (4.3)
Age—50–54	91 (2.3)
Age—55–59	62 (1.6)
Age—60–64	30 (0.8)
Age—65–69	10 (0.3)
Age—70+	4 (0.1)
Highest education—Junior high school	580 (14.9)
Highest education—High school	1632 (41.8)
Highest education—Technical/Vocational	602 (15.4)
Highest education—University	1022 (26.2)
Highest education—Graduate school	64 (1.6)
Employment—Employed	3699 (94.8)
Employment—Unemployed	201 (5.2)
History of mental disorders—Yes	568 (14.6)
Past‐Year Cannabis User—Yes	2981 (76.4)
First Cannabis Use—< 15	128 (3.3)
First Cannabis Use—15–19	1956 (50.2)
First Cannabis Use—20–24	1458 (37.4)
First Cannabis Use—25–29	253 (6.5)
First Cannabis Use—30–34	64 (1.6)
First Cannabis Use—35–39	19 (0.5)
First Cannabis Use—40–44	12 (0.3)
First Cannabis Use—45–49	7 (0.2)
First Cannabis Use—50–54	1 (0.0)
First Cannabis Use—55–59	2 (0.0)
Tobacco use—Yes	3657 (93.8)
Alcohol use—Yes	3755 (96.3)
Methamphetamine use—Yes	405 (10.4)
Organic solvent use—Yes	220 (5.6)
Cocaine use—Yes	400 (10.3)
Natural hallucinogen use—Yes	509 (13.1)
Other hallucinogen use—Yes	753 (19.3)
Opioid use—Yes	26 (0.7)
Benzodiazepine hypnotic use—Yes	260 (6.7)
OTC medication use—Yes	575 (14.7)
Other drug use—Yes	531 (13.6)

*Note:* This table summarizes demographic variables (gender, age, education level, employment status), history of mental disorders, age of first cannabis use, and self‐reported lifetime use of cannabis and other substances among community‐based cannabis users in Japan.

### 3.2 Transition Patterns of Cannabis and Other Substances

**FIGURE 1:**
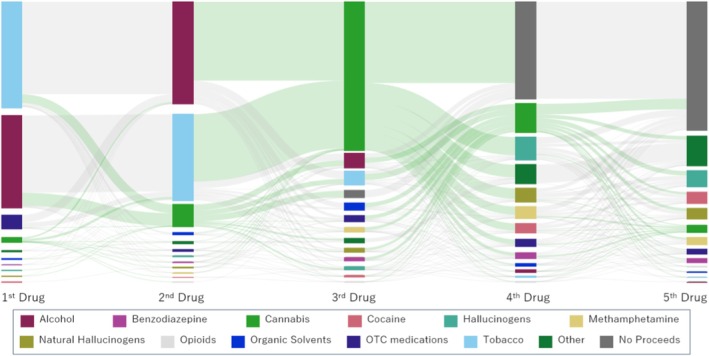
Sankey diagram illustrating the sequential patterns of substance use among community‐based cannabis users in Japan (*N* = 3900). The diagram displays the progression from the first to the fifth substance used, including cannabis. The width of each flow represents the number of respondents reporting a given transition. Tobacco (48.1%) and alcohol (42.3%) were the most common initial substances, followed by benzodiazepines and cannabis. Cannabis was most frequently used as the third substance (68.2%). Notably, 54.7% of individuals who used cannabis as their third substance reported no further transition to other substances. This visualization highlights that cannabis use in Japan typically follows the use of alcohol and tobacco and does not commonly lead to the use of other substances.

The Sankey diagram (Figure 1) illustrates the sequential patterns of substance use, including cannabis. Tobacco (*n* = 1876, 48.1%) was the most common first substance, followed by alcohol (*n* = 1648, 42.3%), benzodiazepines (*n* = 241, 6.2%), and cannabis (*n* = 88, 2.3%). As for the second substance, alcohol was most frequently reported (*n* = 1827, 46.8%), followed by tobacco (*n* = 1535, 39.4%), cannabis (*n* = 397, 10.2%), and organic solvents (*n* = 39, 1.0%).

For the third substance, cannabis was the most frequently reported (*n* = 2661, 68.2%), followed by alcohol (*n* = 258, 6.6%), tobacco (*n* = 240, 6.2%), “no transition” (*n* = 131, 3.4%), and organic solvents (*n* = 122, 3.1%). Regarding the fourth substance, “no transition” (*n* = 1737, 44.5%) was the most common, followed by cannabis, hallucinogens, and others. For the fifth substance, “no transition” (*n* = 2295, 58.8%) remained the most common, followed by others, hallucinogens, cocaine, and natural hallucinogens.

Overall, many cannabis users had prior experience with alcohol or tobacco before initiating cannabis use. Among those who used cannabis as their third substance (*n* = 2661), 1455 (54.7%) did not proceed to use any other substances.

### 3.3 Odds and Odds Ratio of Transitioning to Other Substances After Cannabis Use

The odds and odds ratio (OR) for using major substances after cannabis use are presented in Tables 2, 3, 4, 5. The odds of respondents using other substances after cannabis use were 1.24 for alcohol, 0.77 for tobacco, 0.08 for methamphetamine, and 0.78 for any illegal drug. Logistic regression analysis identified multiple factors as being significantly associated with an increased likelihood of transitioning to specific substances after cannabis use. An OR greater than 1 indicates increased odds of transitioning to each substance relative to the reference group, whereas an OR less than 1 suggests a decreased likelihood. ORs were statistically significant for alcohol, being age 50 or older (OR = 0.23) (Table 2); for tobacco, being male (OR = 2.20), being age 50 or older (OR = 0.18), and having a history of mental health disorders (OR = 2.17) (Table 3); for methamphetamine, being age 30 or older (with particularly elevated odds ratios observed among those age 40 and above), and having completed only junior high school as the highest level of education (OR = 0.34 compared to high school, 0.28 compared to technical/vocational school, 0.19 compared to university, 0.22 for graduate school) (Table 4); for any illegal drug, being in the age groups of 40–54 years, with the highest education being high school or technical/vocational school (Table 5). Hosmer and Lemeshow's goodness‐of‐fit test *p* values were not significant for any of the logistic regression analyses.

**TABLE 2:** Result of logistic regression analysis for transcending to alcohol after experiencing cannabis.

Predictor (*n* = 322, Odds = 1.24)	OR	Log(OR) SE	95% CI (lower)	95% CI (upper)	*p*
Intercept	0.93	0.48	0.36	2.42	0.87
Gender: Male	1.59	0.30	0.89	2.85	0.12
Age: 20 ~ 24	1.02	0.44	0.43	2.38	0.96
Age: 25 ~ 29	0.98	0.47	0.39	2.45	0.97
Age: 30 ~ 34	0.92	0.48	0.35	2.35	0.86
Age: 35 ~ 39	0.84	0.48	0.32	2.17	0.72
Age: 40 ~ 44	0.72	0.56	0.24	2.15	0.56
Age: 45 ~ 49	0.38	0.73	0.08	1.57	0.19
Age: 50 ~	0.23	0.65	0.06	0.78	0.02
Highest Education: High school	1.33	0.33	0.70	2.53	0.38
Highest Education: Technical/Vocational	1.22	0.42	0.54	2.80	0.63
Highest Education: University or higher	1.03	0.37	0.50	2.15	0.93
Employment: Unemployed	0.92	0.44	0.39	2.20	0.84
History of mental disorders: Yes	0.75	0.31	0.41	1.37	0.34

*Note:* Hosmer and Lemeshow goodness‐of‐fit test *p* = 0.6562. Reference group: Female for gender, 19 or younger for age, junior high school for highest education, employed for employment status, no for history of mental disorders. Subsample includes cannabis users who did (*n* = 178) or did not (*n* = 144) transition to alcohol use, excluding those who experienced alcohol before cannabis.

*Abbreviations:* CI: Confidence Interval; OR: Odds Ratio; SE: Standard Error.

**TABLE 3:** Result of logistic regression analysis for transcending to tobacco after experiencing cannabis.

Predictor (*n* = 393, Odds = 0.77)	OR	Log(OR) SE	95% CI (lower)	95% CI (upper)	*p*
Intercept	0.78	−0.25	0.31	1.93	0.58
Gender: Male	2.20	0.79	1.36	3.65	0.00
Age: 20 ~ 24	0.84	−0.18	0.38	1.87	0.67
Age: 25 ~ 29	0.60	−0.51	0.25	1.45	0.26
Age: 30 ~ 34	0.71	−0.34	0.29	1.72	0.45
Age: 35 ~ 39	0.82	−0.20	0.31	2.17	0.69
Age: 40 ~ 44	0.89	−0.11	0.24	3.20	0.86
Age: 45 ~ 49	0.58	−0.55	0.15	2.05	0.40
Age: 50 ~	0.18	−1.73	0.03	0.72	0.02
Highest Education: High school	0.77	−0.26	0.35	1.68	0.51
Highest Education: Technical/Vocational	0.70	−0.36	0.28	1.71	0.44
Highest Education: University or higher	0.57	−0.57	0.25	1.27	0.17
Employment: Unemployed	0.64	−0.45	0.27	1.43	0.28
History of mental disorders: Yes	2.17	0.77	1.21	3.95	0.01

*Note:* Hosmer and Lemeshow goodness‐of‐fit test *p* = 0.05942. Reference group: Female for gender, 19 or younger for age, junior high school for highest education, employed for employment status, no for history of mental disorders. Subsample includes cannabis users who did (*n* = 171) or did not (*n* = 222) transition to tobacco use, excluding those who experienced tobacco before cannabis.

*Abbreviations:* CI: Confidence Interval; OR: Odds Ratio; SE: Standard Error.

**TABLE 4:** Result of logistic regression analysis for transcending to methamphetamine after experiencing cannabis.

Predictor (*n* = 3769, Odds = 0.08)	OR	Log(OR) SE	95% CI (lower)	95% CI (upper)	*p*
Intercept	0.09	0.32	0.05	0.16	0.00
Gender: Male	1.01	0.18	0.72	1.44	0.95
Gender: Other	0.50	1.07	0.03	2.72	0.52
Age: 20 ~ 24	0.82	0.34	0.43	1.63	0.55
Age: 25 ~ 29	1.46	0.34	0.77	2.91	0.26
Age: 30 ~ 34	2.40	0.33	1.30	4.69	0.01
Age: 35 ~ 39	2.57	0.33	1.36	5.11	0.00
Age: 40 ~ 44	5.95	0.32	3.23	11.62	0.00
Age: 45 ~ 49	8.82	0.35	4.53	18.01	0.00
Age: 50 ~ 54	7.69	0.41	3.43	17.35	0.00
Age: 55 ~ 59	3.99	0.53	1.33	10.78	0.01
Age: 60 ~	6.69	0.51	2.32	17.93	0.00
Highest Education: High school	0.34	0.17	0.25	0.47	0.00
Highest Education: Technical/Vocational	0.28	0.21	0.19	0.43	0.00
Highest Education: University	0.19	0.21	0.13	0.29	0.00
Highest Education: Graduate school	0.22	0.54	0.07	0.58	0.01
Employment: Unemployed	1.07	0.28	0.60	1.80	0.82
History of mental disorders: Yes	1.36	0.17	0.96	1.89	0.08

*Note:* Hosmer and Lemeshow goodness‐of‐fit test *p* = 0.1877. Reference group: Female for gender, 19 or younger for age, junior high school for highest education, employed for employment status, no for history of mental disorders. Subsample includes cannabis users who did (*n* = 274) or did not (*n* = 3495) transition to methamphetamine use, excluding those who experienced methamphetamine before cannabis.

*Abbreviations:* CI: Confidence Interval; OR: Odds Ratio; SE: Standard Error.

**TABLE 5:** Result of logistic regression analysis for transcending to any illegal drug after experiencing cannabis.

Predictor (*n* = 3432, Odds = 0.78)	OR	Log(OR) SE	95% CI (lower)	95% CI (upper)	*p*
Intercept	0.76	0.16	0.55	1.04	0.09
Gender: Male	1.04	0.09	0.86	1.25	0.69
Gender: Other	0.76	0.46	0.30	1.83	0.55
Age: 20 ~ 24	1.05	0.15	0.79	1.40	0.74
Age: 25 ~ 29	1.21	0.16	0.89	1.64	0.23
Age: 30 ~ 34	1.15	0.16	0.84	1.58	0.38
Age: 35 ~ 39	1.35	0.17	0.98	1.88	0.07
Age: 40 ~ 44	1.96	0.18	1.39	2.78	0.00
Age: 45 ~ 49	3.02	0.23	1.93	4.77	0.00
Age: 50 ~ 54	2.15	0.28	1.25	3.76	0.01
Age: 55 ~ 59	1.23	0.32	0.65	2.29	0.52
Age: 60 ~	2.06	0.37	1.00	4.32	0.05
Highest Education: High school	0.70	0.11	0.56	0.86	0.00
Highest Education: Technical/Vocational	0.72	0.13	0.56	0.94	0.01
Highest Education: University	0.88	0.12	0.70	1.11	0.27
Highest Education: Graduate school	0.77	0.30	0.42	1.39	0.38
Employment: Unemployed	0.91	0.16	0.66	1.26	0.59
History of mental disorders: Yes	1.09	0.10	0.88	1.33	0.43

*Note:* Hosmer and Lemeshow goodness‐of‐fit test *p* = 0.5991. Reference group: Female for gender, 19 or younger for age, junior high school for highest education, employed for employment status, no for history of mental disorders. Subsample includes cannabis users who did (*n* = 1502) or did not (*n* = 1930) transition to any illegal drugs use, excluding those who experienced any illegal drugs before cannabis.

*Abbreviations:* CI: Confidence Interval; OR: Odds Ratio; SE: Standard Error.

## 4 Discussion

This study is considered one of the largest epidemiological surveys conducted among community‐based drug users in Japan. Below, we discuss the key findings derived from the present analysis.

### 4.1 Does Cannabis Use Lead to the Use of Other Substances?

While this survey targeted individuals with a history of cannabis use, over 90% of respondents reported prior experience with alcohol and tobacco. Most participants identified alcohol or tobacco as the first substance they used, with cannabis typically appearing as the third most common substance. Based on this trend, if one were to classify substances by their role as “gateway drugs,” alcohol and tobacco would arguably precede cannabis in that function. Additionally, odds calculated for the transition to other drug use after cannabis initiation were low. Specifically, the odds for methamphetamine use—one of the most problematic illegal drugs in Japan—were only 0.08, suggesting that the majority of cannabis users did not progress to methamphetamine use. Even when considering the broader category of illegal drugs, including substances like MDMA, the odds remained below 1 (0.78), indicating that most cannabis users did not proceed to use other illegal substances. These findings challenge the notion that cannabis alone should be labeled a “gateway drug,” as such a claim appears scientifically unsupported within the Japanese context.

Logistic regression analysis revealed that being age 30 or older and having completed only junior high school were associated with an increased likelihood of transitioning from cannabis to methamphetamine use. These findings suggest that broader sociocultural and generational factors, rather than cannabis use itself, may play a significant role in the progression to stimulant use.

Individuals aged 30 and older may have grown up in an era when methamphetamine use was more prevalent and methamphetamine was more readily accessible in certain subcultural or socioeconomic contexts. In Japan, methamphetamine has had a long history of use, particularly during the postwar period and into the late 20th century, with periodic resurgences in its popularity among specific populations. Older individuals in our sample may therefore have been more exposed to drug use environments that included methamphetamine as a more normalized or accessible substance [27]. Additionally, having a lower educational background, such as completing only junior high school, may reflect structural vulnerabilities, including reduced access to accurate drug‐related information, limited health literacy, and a higher probability of socioeconomic disadvantage. These factors are known to be associated with increased risk for substance use disorders and may contribute to reduced social support, unstable employment, and greater exposure to high‐risk peer groups or communities.

Rather than implying a causal gateway effect of cannabis use, these results highlight the importance of considering the broader life context in which substance use occurs. Social determinants such as age cohort, educational background, and socioeconomic position appear to shape patterns of substance progression independently of the pharmacological properties of cannabis. Future research should aim to incorporate longitudinal data and more detailed indicators of social environment in order to better understand the mechanisms underlying these transitions.

### 4.2 Alternative Explanations to the Gateway Hypothesis

That said, our survey revealed that 10.4% of cannabis users reported experience with methamphetamine, a figure considerably higher than the estimated lifetime methamphetamine use prevalence of 0.5% in the general Japanese population [28]. This suggests that cannabis users may indeed have higher exposure to other illegal drugs, though this does not establish causality.

The “common liability theory” has been proposed to explain such patterns [29]. According to this theory, the observed order and relationship between substances result not from one drug directly leading to another, but from shared underlying factors—such as genetic, psychological, and social influences—that predispose individuals to multiple substance use. In Japan, strict cannabis regulations may contribute to a situation where cannabis and other illegal drugs circulate within the same black market, increasing users' exposure to various substances. Thus, it may be the regulatory environment, rather than the pharmacological properties of cannabis itself, that creates a “gateway” effect. This notion is further supported by the relatively lower rates of use of legal substances, such as benzodiazepines and prescription drugs, in our sample.

Japan's revision of the Cannabis Control Act in December 2024 introduced harsher penalties for cannabis possession, raising sentences from up to 5–7 years of imprisonment, and for the first time, penalized use itself, allowing criminal charges based on positive urine tests [21]. Although this survey was conducted prior to these legal changes, past experiences suggest that stricter enforcement may further drive cannabis markets underground [30, 31, 32], potentially increasing exposure to other illegal substances. However, no empirical research currently exists to assess how these legal changes will affect drug markets or user behavior. Our findings highlight the urgent need for scientific monitoring to determine whether the revised law truly contributes to public health and welfare.

### 4.3 Limitations

This study has several limitations. First, recruitment relied on self‐selection through online platforms, specifically through Green Zone Japan's social media channels, which advocate for medical cannabis. Therefore, selection bias may have favored respondents with a more favorable attitude toward cannabis and possibly more critical views of other drugs. Additionally, drug use history was self‐reported without external validation.

Second, this study focused solely on cannabis users, limiting the generalizability of results to the broader population. It is possible that a similar survey targeting users of other drugs might yield different patterns.

Third, recall bias cannot be excluded, as participants reported past drug use and its sequence retrospectively.

Finally, in order to ensure anonymity, no personal information, such as email addresses, was collected in this survey. Consequently, duplicate responses were excluded based on self‐reported information. However, this method may not have been sufficient to fully eliminate all duplicate entries. Since no incentives were offered for participation, the likelihood of a single individual submitting multiple responses was considered to be low, and any influence from duplicate responses was assumed to be minimal.

Despite these limitations, this remains one of the largest and most significant studies on community‐based cannabis users in Japan to date. To overcome these limitations, future large‐scale cohort studies involving the general population will be essential.

## 5 Conclusion

In this additional analysis, we observed that cannabis tends to be the third most commonly used substance, following alcohol and tobacco. The odds of progressing to methamphetamine or other illegal drug use after cannabis initiation were low. Based on the results of this nonrandomized, self‐selected sample of community cannabis users in Japan, we did not observe patterns that support the gateway drug hypothesis.

## Author Contributions

Y.M. initiated the study and drafted the study design and the online survey together with T.S., N.M., Y.A., F.U., I.T., H.M., C.O., T.M., T.A., and T.M. T.S. collected the data, and M.K. performed the statistical analysis. Y.M. and M.K. cowrote the introduction, methods, results, discussion, and conclusion sections. All authors reviewed and approved the final manuscript.

## Ethics Statement

Approval of the Research Protocol by an Institutional Review Board:

This study was approved by the Ethical Review Committee of the National Center of Neurology and Psychiatry (Approval Number: A2020‐117; Approval Date: January 8, 2021).

## Consent

Informed consent was obtained from all participants, and anonymity was preserved.

## Conflicts of Interest

The authors declare no conflicts of interest.

## Supporting information


**Data S1.** Supporting Information.

## Data Availability

The data that supports the findings of this study are available in the supplementary material of this article.
